# Subchondral insufficiency fracture of the knee: progress in the pathogenesis and treatment

**DOI:** 10.3389/fsurg.2025.1640316

**Published:** 2025-10-21

**Authors:** Ziyue Wang, Yi Zhang, Jiale Yuan, Chao Wang, Bangjian He, Hang Pei

**Affiliations:** 1 Department of Orthopedics, Fenghua District Traditional Chinese Medicine Hospital of Ningbo, Ningbo, Zhejiang, China; 2 Department of Orthopedics, The First Affiliated Hospital of Zhejiang Chinese Medical University, Hangzhou, China; 3 Department of Orthopedics, Zhejiang Chinese Medical University, Hangzhou, Zhejiang, China; 4 Department of Orthopedics, Anji County Hospital of Chinese Medicine, Huzhou, Zhejiang, China

**Keywords:** subchondral insufficiency fracture of the knee, subchondral insufficiency fracture, spontaneous osteonecrosis of the knee, subchondral lesion, review

## Abstract

Subchondral Insufficiency Fracture of the Knee (SIFK) is a disease with unclear pathogenesis and rapid progression, it is prone to developing end-stage osteoarthritis. Due to the lack of a definitive treatment strategy for SIFK in the clinic, knee replacement is often the preferred measure. However, in recent years, as the disease has become better understood, more and more effective treatments have been proposed, but there is a lack of summarization. Therefore, this article summarizes its pathogenesis and therapeutic advances.

## 1 Introduction

**Figure 1:**
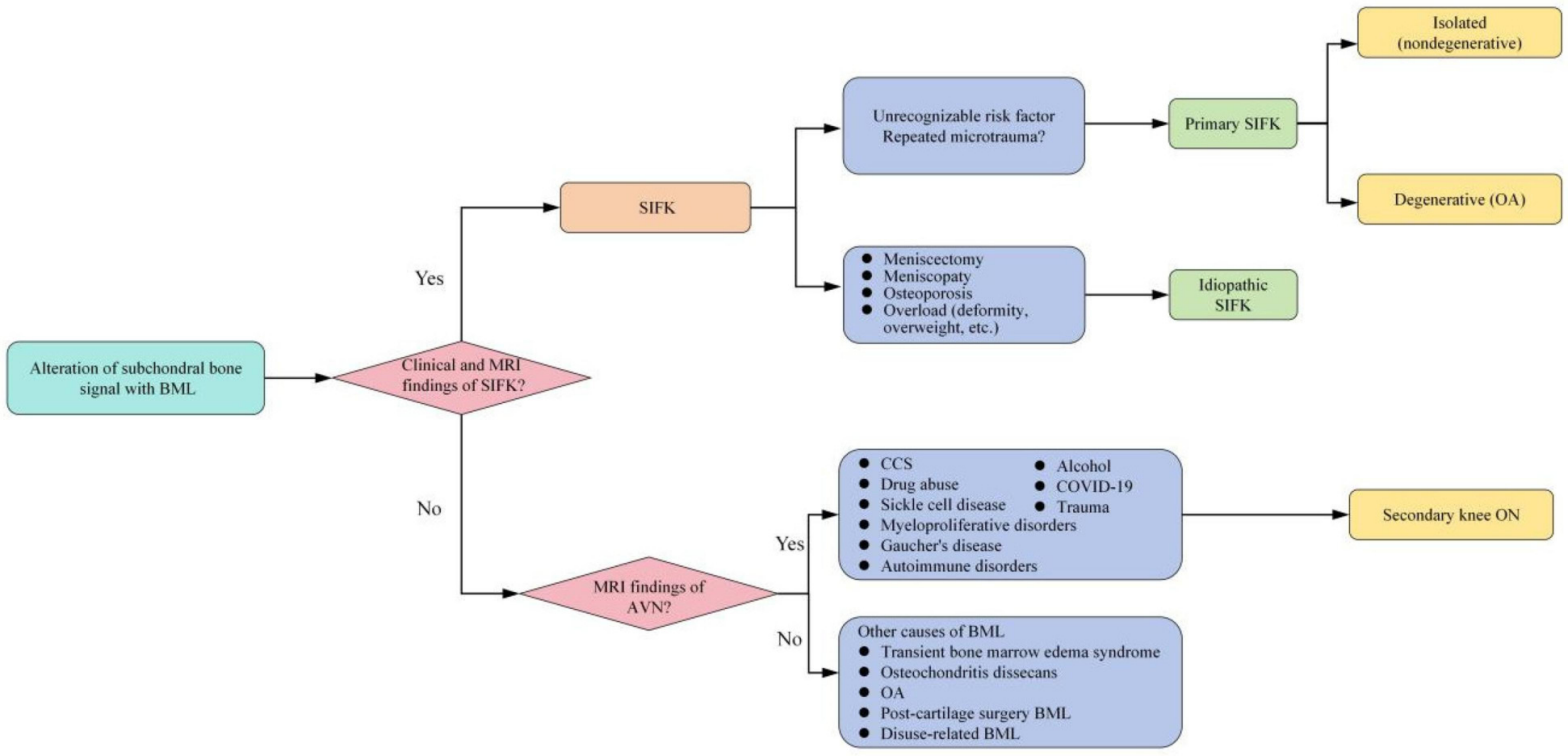
New proposed classification of knee BMLs. AVN, avascular necrosis; BMD, bone mineral density; BML, bone marrow lesions; CCS, corticosteroids; MRI, magnetic resonance imaging; OA, osteoarthritis; ON, osteonecrosis; SIFK, subchondral insufficiency fracture of the knee.

**Figure 2:**
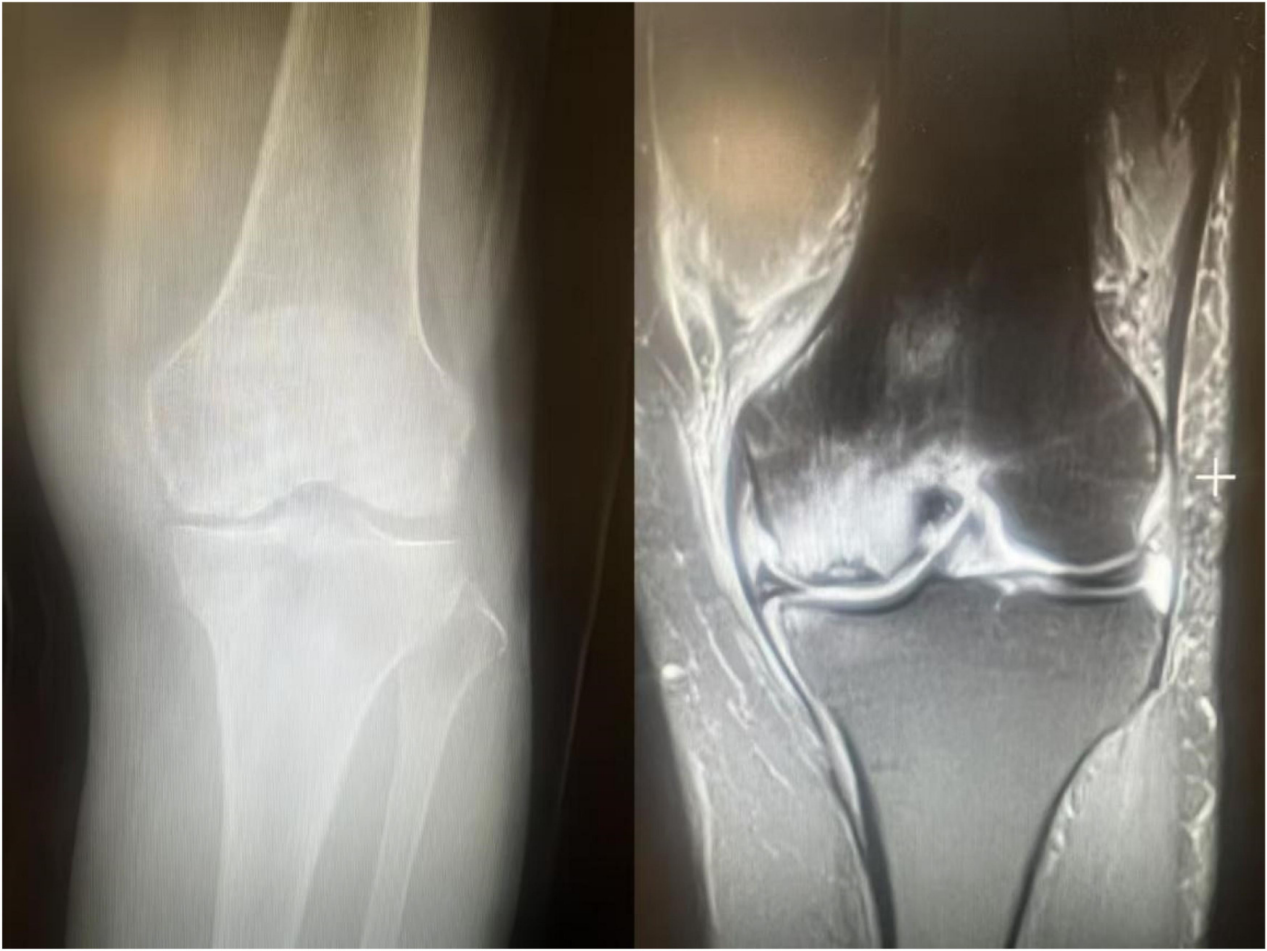
Coronal position of preoperative x-ray and MRI in patients with SIFK.

Subchondral insufficiency fracture of the knee (SIFK) was initially termed spontaneous osteonecrosis of the knee (SONK), a nomenclature first proposed by Ahlbäck et al. in 1968 (1). However, their study failed to provide histological evidence supporting osteonecrosis. It was not until 2000 that Yamamoto and Bulloug, through analysis of surgical bone specimens from SONK patients, demonstrated that the histological features were inconsistent with osteonecrosis (2). Subsequent studies further corroborated these findings, establishing that the pathological entity was fundamentally a subchondral fracture (3). Based on this evidence, Za P's team synthesized prior research to propose a new framework distinguishing SIFK from osteonecrosis of the knee (4) (Figure 1), advocating for the preferential use of “SIFK” over “SONK”. SIFK most commonly occurs in the medial femoral condyle of the knee (Figure 2), though involvement of the lateral condyle and tibial plateau has also been documented (5). The condition progresses rapidly, often leading to articular surface collapse and subsequent advanced SIFK. Epidemiological studies indicate a prevalence as high as 9.6% among elderly individuals aged 65 and older, with women affected 3–5 times more frequently than men (6). Additionally, the condition is increasingly observed in young adults engaged in excessive physical activity (7).

The pathophysiology of SIFK remains incompletely elucidated, and existing studies lack systematic synthesis. Both conservative treatment plans and surgical interventions for this condition have demonstrated diversified development with notable therapeutic efficacy. However, a clinical misconception persists regarding immediate arthroplasty upon diagnosis, and standardized treatment guidelines have yet to be established. In recent years, with the identification of novel risk factors and innovations in therapeutic approaches, this review aims to provide clinicians with a comprehensive synthesis of SIFK's pathophysiology and treatment strategies.

## 2 SIFK: pathogensis

**Figure 3:**
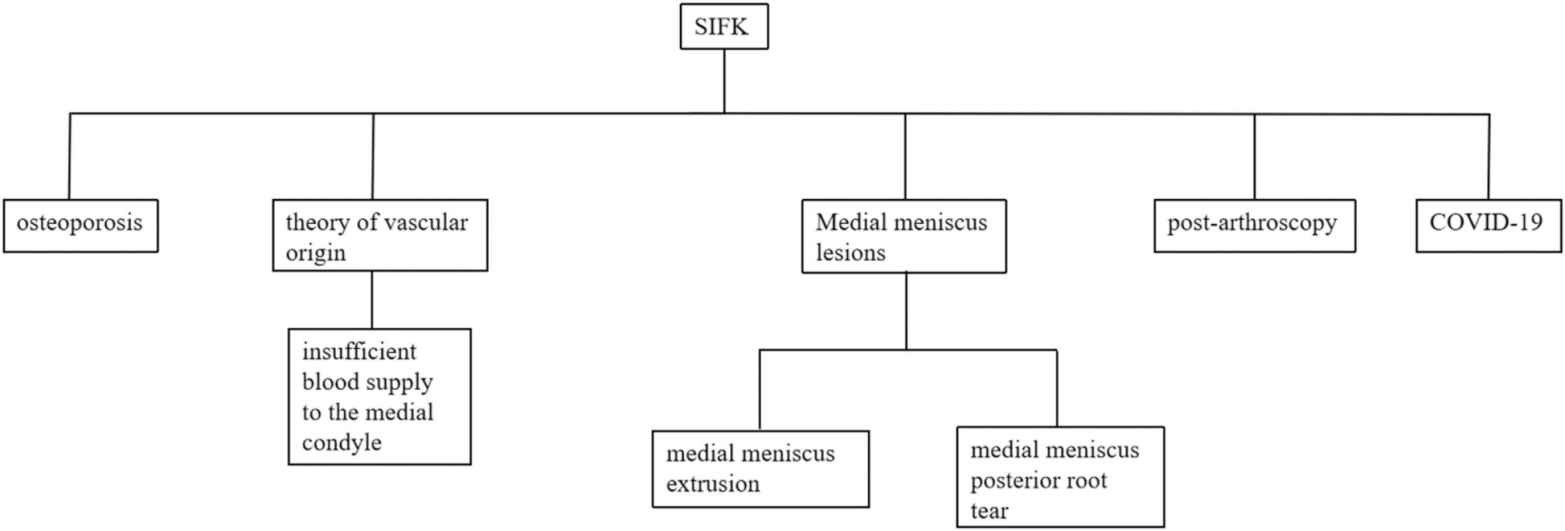
Pathogenesis of SIFK.

The pathogenesis of SIFK is detailed in Figure 3.

### 2.1 Osteoporosis

The medial femoral condyle of the knee joint bears higher stress than the lateral condyle (8), making it more susceptible to subchondral insufficiency fracture of the knee (SIFK) under prolonged stress loading, particularly in patients with compromised bone quality (2, 9, 10). Akamatsu et al. (11) compared the bone mineral density (BMD) of femoral condyles and tibial plateaus between 26 elderly female SIFK patients and osteoarthritis (OA) patients, revealing significantly lower BMD values in the SIFK group. Furthermore, Horikawa's team (12) employed magnetic resonance imaging (MRI) and dual-energy x-ray absorptiometry (DXA) to evaluate bilateral knees in SIFK patients, demonstrating markedly reduced BMD in affected femoral condylar regions compared to contralateral unaffected sites. These findings indicate a strong association between SIFK pathogenesis and decreased BMD. Therefore, in clinical practice, systematic BMD assessment and standardized anti-osteoporosis treatment should be considered essential components of SIFK prevention and management for high-risk populations (e.g., elderly women and patients with chronic medial knee pain).

### 2.2 Theory of vascular origin

Anatomical studies have revealed that the intraosseous vessels of the medial femoral condyle penetrate to a shallower depth compared to those of the lateral condyle (13, 14). Furthermore, the subchondral bone of the medial condyle is predominantly supplied by a single terminal intraosseous branch of the superior medial genicular artery (15). Damage to this sole nutrient pathway can severely compromise vascular perfusion. Specifically, vascular occlusion (e.g., due to microthrombi or atherosclerosis) directly reduces blood inflow, while post-traumatic bone marrow edema elevates intraosseous pressure. This increase in intraosseous pressure further compresses the fragile vessels within the bone marrow, exacerbating ischemia and establishing a vicious cycle that severely disrupts the microcirculation. When combined with prolonged mechanical loading, these factors are prone to facilitate the development of SIFK. Through immunohistochemical analysis, Lankes M et al. (16) revealed that the intraosseous vascular pathways supplying the lateral portion of the medial condyle exhibit significantly longer trajectories and markedly reduced vascular density. The authors therefore proposed that compromised vascular supply to the medial condyle may represent one of the pathogenic mechanisms underlying SIFK.

### 2.3 Medial meniscus lesions

The meniscus is an integral part of the complex biomechanical system of the knee. It provides load bearing by increasing the contact area and providing an evenly distributed articular surface, which is essential in load sharing, and the medial meniscus shares more pressure (17). Therefore, medial meniscus lesions are considered to be a risk factor for the creation and development of SIFK.

#### 2.3.1 Extrusion of the medial meniscus

**Figure 4:**
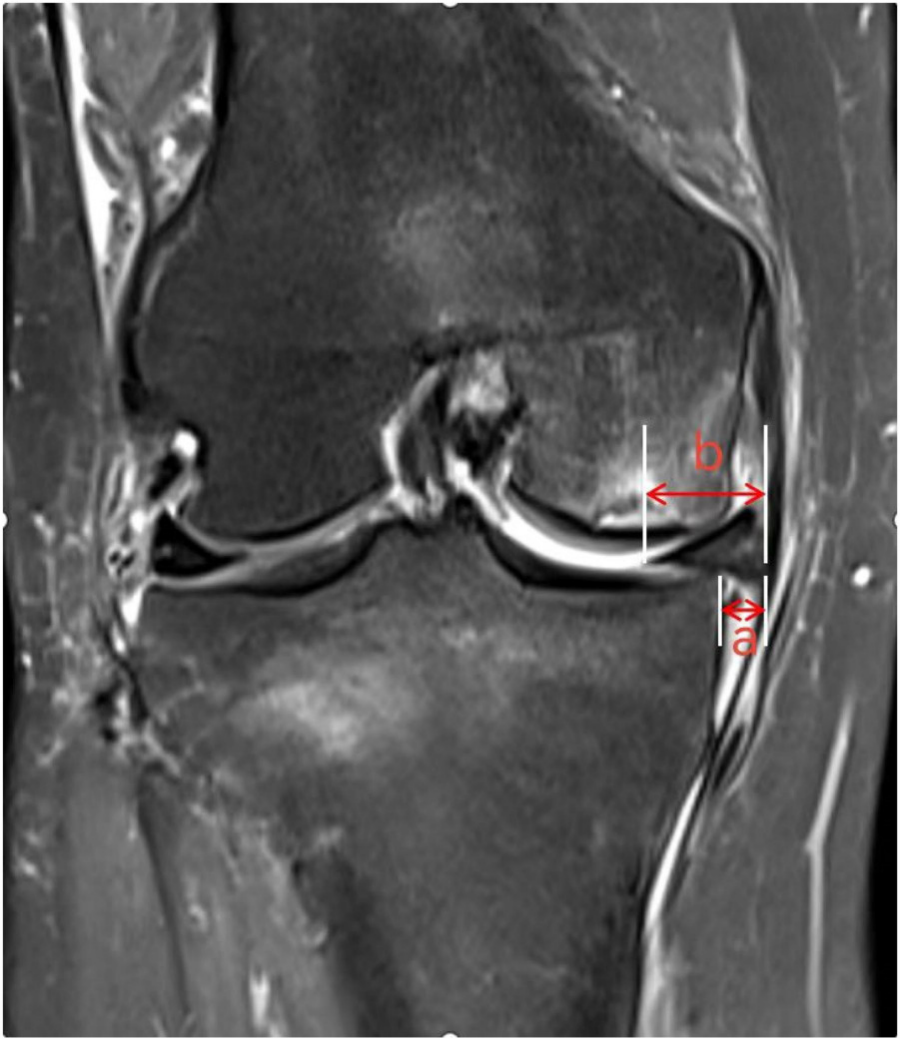
The distance from the outer edge of the medial tibial plateau to the outer edge of the medial meniscus is measured on the image, with the measured meniscal protrusion value denoted as “a”. The absolute value of medial meniscal protrusion is denoted as “b”. The relative protrusion rate of the medial meniscus is calculated as a/b × 100%.

Meniscal extrusion (ME) refers to a pathological condition where the meniscal margin extends beyond the tibial plateau edge, resulting from the disruption of the internal circumferential collagen fiber structure that maintains hoop strength. In their review, Papalia GF et al. reported that ME is closely associated with articular cartilage wear and the development of OA, and may serve as a predictive risk factor and an imaging diagnostic marker for early-stage OA (18). The biomechanical dysfunction of the knee joint induced by ME (e.g., altered load distribution, reduced stability) accelerates the degradation of articular cartilage matrix, ultimately promoting the development of OA and SIFK (19). Shuhei Oda's team (20) conducted an MRI analysis of 108 SIFK patients and identified medial meniscal extrusion in 39 cases (36.1%), with the degree of extrusion positively correlated with SIFK severity. Based on these findings, the researchers proposed that medial meniscal extrusion is a critical pathogenic mechanism in the development and progression of SIFK. Furthermore, Hashimoto et al. suggested that when the relative extrusion percentage of the medial meniscus (i.e., the ratio of the extruded meniscal width beyond the tibial plateau edge to the total meniscal width, Figure 4) exceeds 33%, patients exhibit a significantly higher risk of disease progression (8). ME disrupts normal knee biomechanics, particularly by exacerbating stress concentration in the medial compartment, thereby playing a pivotal role in the multifactorial pathogenic cascade of SIFK involving “biomechanics-bone metabolism-microenvironment” interactions. In clinical practice, routine measurement of the relative extrusion percentage of the medial meniscus in SIFK patients—combined with comprehensive assessments including bone mineral density, symptomatology, and imaging staging—provides critical guidance for risk stratification and the formulation of individualized treatment plans.

#### 2.3.2 Medial meniscus posterior root tears (MMPRT)

MMPRT is a clinically significant injury that induces symptoms comparable to those of total meniscectomy (21). Yang Q et al. (22) demonstrated through gait kinetic analysis that MMPRT leads to focal stress elevation in both the medial and lateral femoral condyles, which may accelerate joint degeneration. Yamagami R et al. (23) conducted a comparative MRI analysis of 45 SIFK patients and 105 OA patients, confirming a stronger association between SIFK and MMPRT. In a systematic review by Hussain ZB et al., 12 out of 26 included studies identified meniscal tears as a contributing pathophysiology of SIFK (24). Additionally, due to the elastic properties of the meniscus, differential extrusion occurs between unloaded and loaded states, termed dynamic extrusion (25). This physiological dynamic behavior is critical for meniscal function, facilitating shock absorption and load distribution (26). Papalia GF's review reported that, compared to intact menisci, MMPRT significantly attenuates dynamic meniscal extrusion, resulting in the “dead meniscus sign”, which disrupts load distribution across the knee joint and contributes to the development of SIFK and knee osteoarthritis (KOA) (18). Based on this evidence, orthopedic surgeons should fully recognize the pathological relationship between MMPRT and SIFK and implement timely interventions to prevent the onset and progression of SIFK.

### 2.4 Post-arthroscopy

With the widespread application of knee arthroscopy, increasing attention has been drawn to the complication known as post-arthroscopic osteonecrosis of the knee (PAONK). However, a systematic review by Za P et al. (27) reported that although PAONK and SONK exhibit considerable overlap in clinical symptoms and radiological characteristics, they represent distinct disease entities. To evaluate and synthesize the histopathological features of PAONK cases, the same team conducted a scoping review. They reported that the most common histological finding was SIF, observed in 94.1% of cases, and suggested that the histological evidence of PAONK is more consistent with SIF rather than true osteonecrosis (28).

Although mechanisms such as mechanical trauma, elevated intra-articular pressure, or thermal injury induced by arthroscopy have been proposed (29–31), the notion that arthroscopic procedures directly cause SIFK has been questioned in recent years. Current evidence supports the following interpretations: a. Preexisting meniscal injury and occult SIFK: Unrecognized SIFK may already be present prior to arthroscopy. Due to the diagnostic window period of MRI, early-stage SIFK may not be visible on preoperative imaging (27). b. Altered biomechanics following meniscectomy: Excessive or improperly performed meniscectomy is considered a key factor leading to postoperative biomechanical changes. Kobayashi et al. (32) compared preoperative and postoperative MRI findings in patients who underwent arthroscopic meniscectomy and found bone marrow signal abnormalities in 34% of patients postoperatively. Further research by Fukui et al. (33) demonstrated significant height discrepancies and heterogeneous sclerosis in the resected medial meniscus after arthroscopic meniscectomy, with altered mechanical environments promoting the occurrence of SIF. In summary, it should be recognized that arthroscopic procedures may act as contributing factors rather than direct causes of SIFK. However, this conceptual paradigm still faces challenges. The primary issue lies in the timeliness of diagnosis: due to the limitations of the MRI diagnostic window, it is difficult to confirm the presence of occult SIFK preoperatively, which complicates etiological attribution. Therefore, high-quality RCTs should be conducted, incorporating multimodal preoperative assessments—including high-resolution MRI—in patients scheduled for arthroscopic surgery (particularly meniscectomy), to facilitate earlier identification of individuals at high risk for SIFK. Furthermore, biomechanical engineering techniques such as finite element analysis could be employed to quantitatively simulate the effects of different surgical techniques on internal knee stress, thereby mechanistically elucidating the threshold for SIFK occurrence. Surgeons should also fully appreciate the biomechanical importance of meniscal tissue and shift the surgical philosophy from “resection” to maximal “preservation and repair”, thereby reducing the risk of post-arthroscopic SIFK at its source.

### 2.5 COVID-19

Since COVID-19 can induce vascular endothelial injury and lead to a hypercoagulable state, it may affect nearly all organ systems throughout the body (34). Statistical data indicate that 92.30% of hospitalized COVID-19 patients report musculoskeletal symptoms upon admission (35). Za P et al. conducted a case-based systematic review and reported that COVID-19-infected patients typically develop knee pain after an average of 11 weeks (36). This suggests that COVID-19 may be an independent risk factor for osteonecrosis. It is noteworthy that this association becomes more complex clinically, as critically ill patients are often subjected to corticosteroid therapy. Corticosteroids, which have been widely used as emergency treatment during the COVID-19 pandemic, are themselves clearly associated with the development of spontaneous osteonecrosis (37, 38). A systematic review by Za P et al. reported that patients with a history of COVID-19 infection develop osteonecrosis earlier and require lower doses of corticosteroids, suggesting that osteonecrosis should be considered a component of long COVID syndrome (39). Additionally, Malinowski et al. (40) reported two cases of COVID-19 patients who had not received corticosteroids but exhibited diffuse bone marrow edema in the femoral condyles on MRI. In conclusion, orthopedic surgeons should not underestimate the musculoskeletal symptoms in COVID-19 patients and should conduct long-term follow-up. Persistent joint symptoms warrant prompt MRI evaluation. Given the limited sample sizes in existing studies, higher-quality research with larger cohorts is needed to elucidate the role of COVID-19 in the pathogenesis of osteonecrosis.

## 3 SIFK: treatment

**Figure 5:**
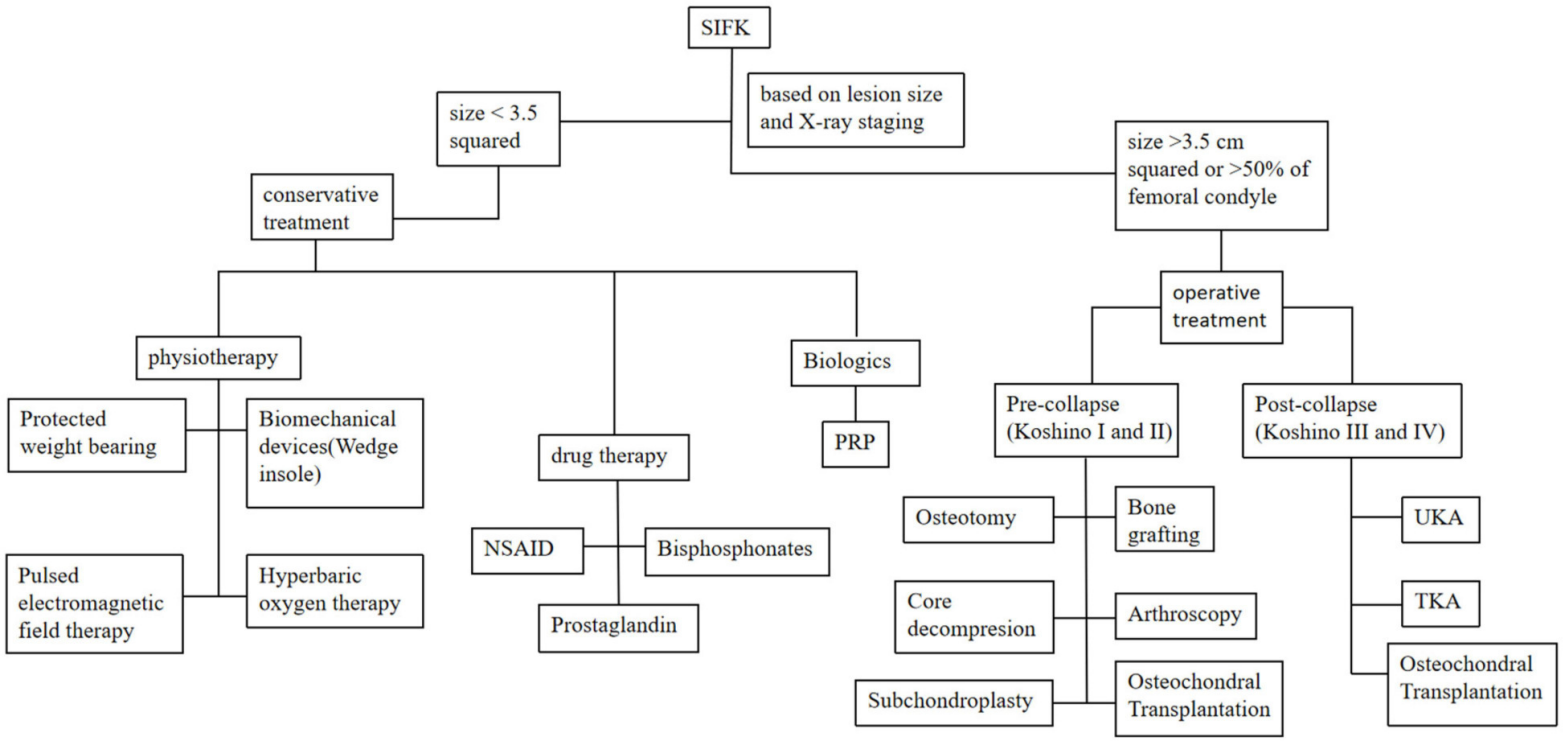
Step therapy strategies of SIFK.

The management of SIFK is typically determined by lesion size and radiographic staging (Koshino classification, Table 1), with options including conservative treatment or surgical intervention (41). Lesion area is calculated by multiplying the maximum width on coronal radiographs by the maximum length on sagittal radiographs. Small lesions (<3.5 cm²) are managed nonoperatively, while medium (3.5–5.0 cm²) and large lesions (>5.0 cm²) generally require surgery. Notably, large lesions frequently lead to femoral condylar collapse, often necessitating total joint arthroplasty (42, 43). Lotke et al. (44) proposed that treatment plans should be based on the extent of femoral condylar involvement. Their study demonstrated that when lesions affected 32% of the medial femoral condyle, only 6 of 23 knees required surgery. In contrast, all patients with lesions involving >50% of the condyle underwent joint replacement (Figure 5: step therapy strategies of SIFK).

**Table 1:** Koshino classification.

Classification	X-ray presentation
Type I	No unusual manifestations
Type II	Oval translucent shadow in the subchondral region or flattening of the femoral condyle
Type III	Increased translucent shadow of the lesion with sclerotic bands or subchondral collapse
Type IV	Significant arthritic manifestations such as osteophyte formation, subchondral bone sclerosis, narrowing or loss of joint space

### 3.1 Conservative treatment

#### 3.1.1 Physiotherapy

##### 3.1.1.1 Protected weight bearing

Protected weight bearing is effective in reducing marrow pressure in lower limb bones (45). Patients with early SIFK can reduce clinical symptoms and gradually return to normal weight bearing after 4–8 weeks of restricted weight bearing (41). Jordan et al. (46) reviewed 40 patients with early SIFK, after 6 weeks of protected weight bearing, 39 patients showed significant improvement in pain and functional scores, and only 1 patient required arthroscopic surgical intervention.

##### 3.1.1.2 Biomechanical devices (wedge insole)

In the 1980s, Sasaki first proposed that wedge-shaped insoles might treat OA by altering knee joint alignment (47). Joint loading during walking is closely associated with the development of knee OA, characterized by uneven pressure distribution between the medial and lateral condyles, with the medial condyle bearing a higher load rate (48). The study by Atoun et al. (49) further corroborated the role of load imbalance in the progression of SIFK, revealing significant bilateral biomechanical asymmetry in SIFK patients, which may lead to long-term adverse outcomes. Research indicates that wedge insoles effectively reduce the knee adduction moment in SIFK patients, ameliorate bilateral biomechanical asymmetry, and consequently alleviate medial condylar loading (50). Based on these biomechanical principles, investigators explored the clinical efficacy of wedge insoles for early-stage SIFK (Koshino stages I–II). After six months of treatment in 17 early-stage SIFK patients, pain severity decreased by 53%, and functional limitations were reduced by 44% (49). These findings demonstrate that wedge insoles address medial compartment load imbalance, offering a viable non-surgical intervention for early SIFK. However, it is noteworthy that the natural progression of SIFK exhibits variability, and whether the observed improvements are solely attributable to wedge insole intervention requires validation through more rigorous large-scale RCTs.

##### 3.1.1.3 Pulsed electromagnetic field therapy (PEMF)

PEMF therapy is a non-invasive treatment. It applies a magnetic field generated by intermittent current pulses to living tissue over a short period of time (51). PEMF can activate signalling pathways and regulate ion channels and the expression of bone-related genes to enhance osteoblast activity and promote regeneration of neural and vascular tissues, thereby accelerating bone formation during bone repair (52). Moreover, PEMF can increase adenosine A2 receptor density in cell membranes of osteoblasts and chondrocytes (53). Adenosine A2 receptors promote coronary circulation through vasodilation and immune cell suppression, thereby protecting tissues from inflammation (54). Marcheggian et al. (55) treated 28 patients with early SIFK with PEMF for 3 months, and 23 of them showed significant improvement in imaging and knee function. PEMF is a cost-effective treatment for patients with early SIFK. However, most of the existing studies of PEMF for the treatment of early SIFK lack a suitable control group, so more and more rigorous controlled studies are needed to verify its efficacy.

##### 3.1.1.4 Hyperbaric oxygen therapy (HBOT)

HBOT has proven to be an effective complementary therapy for the treatment of various clinical and pathological diseases. Based on its physiological effect of increasing tissue oxygenation or improving oxygen bioavailability, it plays a role in promoting angiogenesis and collagen production (56). This has led researchers to conduct many studies on the mechanism of action of HBOT in the treatment of bone diseases. They found that HBOT increased osteoprotegerin (OPG) and alkaline phosphatase (AKP) levels, both of which are important for bone structural remodelling and bone formation (57, 58). Several studies have shown that HBOT can effectively prevent the progression of osteonecrosis and reverse it (59, 60). After Bosco G et al. (61) used HBOT for 50 times on 37 SIFK patients, the functional scores of all patients were improved. And post-treatment MRI showed that marrow edema of the femoral condyle had subsided in all but one patient. As far as the current literature reports, the treatment of osteonecrosis with HBOT alone often requires dozens of treatments. For some patients, this is a significant cost and also makes this monotherapy less compliant due to the multiple time-consuming. However, as a complementary therapy to other treatments or surgeries, HBOT is a non-negotiable option in terms of expense, efficacy and all other aspects.

#### 3.1.2 Drug therapy

##### 3.1.2.1 Nonsteroidal anti-inflammatory drug (NSAID)

NSAIDs are effective in relieving pain and inflammation and are widely used in musculoskeletal disorders (Such as osteoarthritis, rheumatoid arthritis, chronic pain, etc.) (61, 62). Yates' team (63) used NSAID to treat 20 patients with SONK and achieved excellent results. And Maris (64) treated a young female SONK patient with NSAID for 4 weeks, and her clinical symptoms completely disappeared. However, current evidence indicates that NSAIDs only provide short-term symptomatic relief and fail to halt the pathological progression of SIFK. Therefore, they should be considered as adjunctive therapy in the comprehensive management of SIFK, particularly during acute pain episodes, rather than as curative or long-term maintenance treatment. Moreover, the well-documented risks of NSAIDs—including gastrointestinal mucosal injury, cardiovascular events, and renal impairment (65)—necessitate strict adherence to the principle of “the lowest effective dose for the shortest duration” in clinical decision-making. A thorough multisystem risk assessment must be conducted prior to prescribing NSAIDs, especially for elderly patients.

#### 3.1.3 Bisphosphonates

Bisphosphonates, which inhibit osteoclast-mediated bone resorption, are widely used in the treatment of metabolic bone diseases characterized by excessive osteoclast activity. These agents significantly reduce bone resorption rates during vascular remodeling of bone tissue, promote new bone formation, and thereby delay structural bone destruction (66). Theoretically, this anti-bone resorptive property may have potential value in delaying the progression of SIF. However, it must be clearly emphasized that the inclusion criteria in most current clinical studies on bisphosphonate treatment for knee lesions are based on the imaging manifestation of BME. BME is a common imaging feature shared by multiple diseases, including SIFK, transient osteoporosis, trauma, among others, rather than being specific to strictly diagnosed cases of SIFK.

Although large-scale studies specifically targeting SIFK are lacking, several clinical trials involving patients with knee pain accompanied by bone marrow edema have shown promising signals. Shen Z et al. (67) demonstrated that bisphosphonates increase alkaline phosphatase (ALP) levels in patients with subchondral SIFK and reduce surgical intervention rates. Both ibandronate and alendronate have been confirmed to exhibit definitive therapeutic efficacy in SIFK. Beyond these, additional bisphosphonate formulations have shown clinical potential in bone marrow lesions, particularly at high doses and with parenteral administration. The safety and efficacy of high-dose intravenous clodronate in the treatment of knee osteoarthritis and bone marrow edema have been established (68, 69). Frediani et al. (70) demonstrated that patients receiving 200 mg/week of clodronate exhibited significantly greater improvements in bone mineral density and clinical symptoms compared to those receiving 100 mg/week, indicating a positive correlation between cumulative dose and therapeutic effect. Another study by the same group (71) highlighted that prolonged treatment contributes to sustained efficacy. Regarding intravenous neridronate, Varenna et al. (72) demonstrated that a short-term, high-dose regimen (100 mg/dose, 4 doses over 10 days) significantly reduced Visual Analog Scale (VAS) scores (*P* < 0.001) and improved the extent of bone marrow edema (*P* = 0.002). Guiducci et al. (73) found that a long-term maintenance regimen (25 mg/month for 6 years) significantly enhanced bone repair rates without adverse events. Furthermore, a multicenter clinical study suggested that monthly 25 mg neridronate injections may represent the optimal dose for maximal therapeutic effect (74).

By modulating bone metabolism, bisphosphonates demonstrate clear therapeutic value in reducing surgical risks and promoting bone repair. For early-stage cases accompanied by bone marrow edema without collapse, bisphosphonates can significantly delay disease progression, particularly in osteoporotic patients with concurrent bone marrow edema. However, these findings must be interpreted with caution, as the observed efficacy is based on a heterogeneous “bone marrow edema” population. Whether these results can be directly extrapolated to strictly defined SIFK patients remains to be validated through prospective studies that use clearly defined SIFK diagnostic criteria as inclusion conditions. Furthermore, large-scale, long-term randomized controlled trials are still needed to investigate the safety and efficacy differences among various bisphosphonate preparations. In addition, due to the lack of biomarker-guided individualized dosing models, no standardized treatment protocol has been established. In clinical practice, treatment strategies should be formulated based on bone mineral density, severity of bone marrow edema, and drug tolerance, while also balancing long-term bone safety and functional recovery.

##### 3.1.3.1 Prostaglandin (PG)

PG has anti-thrombotic, vasodilatory and anti-proliferative effects (75). Many clinical studies have shown that PG is effective in the treatment of bone marrow edema and ischaemic osteonecrosis (76, 77). Jäger et al. (78) found that patients with early SIFK had significant improvements in pain, function and imaging outcomes after prostaglandin I2 application. However, advanced patients did not benefit.

#### 3.1.4 Biologics (platelet-rich plasma, PRP)

PRP contains multiple growth factors, including platelet-derived growth factor (PDGF), transforming growth factor-β (TGF-β), insulin-like growth factor (IGF), and vascular endothelial growth factor 70 (VEGF70) (79). It has been widely applied in the treatment of musculoskeletal disorders. An immunohistological study demonstrated that PRP upregulates the expression of osteogenic markers such as β-catenin and alkaline phosphatase (AKP), while concurrently reducing serum levels of triglycerides and total cholesterol, thereby ameliorating hypercoagulability to decelerate osteonecrosis progression (80). Current evidence indicates the profound therapeutic efficacy of PRP for osteonecrosis of the femoral head (ONFH) (81). However, it should be noted that SIFK differs from ONFH in terms of pathophysiology, biomechanical environment, and susceptible populations. To date, high-quality clinical studies on PRP therapy for SONK remain scarce, and its efficacy, safety, and optimal treatment plans (e.g., injection timing, dosage, and frequency) urgently require clarification. Given that SIFK is a rapidly progressive condition leading to severe knee joint destruction and functional impairment—with no ideal conservative interventions currently available to halt its progression—exploring the therapeutic potential of PRP-based bioregenerative therapies in this context carries significant clinical implications and demands immediate attention.

#### 3.1.5 Gene therapy

Although no gene therapy for SIFK has yet entered clinical practice, promising potential has been demonstrated in studies on ONFH, which shares similar pathophysiological features (82). Current strategies primarily focus on using viral (e.g., adeno-associated virus) or non-viral vectors to deliver functional genes—such as those encoding vascular endothelial growth factor and bone morphogenetic protein—specifically to the ischemic necrotic area. This enables sustained and highly localized expression of pro-angiogenic and osteogenic factors, thereby fundamentally improving blood supply and promoting bone repair (83). However, translating gene therapy into clinical application for SIFK still faces major challenges, including but not limited to: selection of optimal cell sources and delivery methods, safety and transfection efficiency of gene vectors, determination of the optimal treatment window (prevention, early intervention, or late-stage repair), and the lack of SIFK-specific animal models for efficacy validation. Nonetheless, owing to its precise targeting capability and long-lasting effects, gene therapy remains a highly attractive direction for future interventions in SIFK.

### 3.2 Operative treatment

#### 3.2.1 Arthroscopy

Since the pathological changes in SIFK originate within the bone, arthroscopic joint cleaning of the knee does not alter the course of the disease. However, for patients with suspected internal knee lesions, knee arthroscopy can help to identify knee cartilage injury. At the same time, arthroscopic clearance can improve the symptoms of SIFK in patients with femoral condylar cartilage stripping, free body in joint, and meniscus injury. Akgun I et al. (84) performed arthroscopic microfracture of the knee in 26 patients with SIFK who had failed conservative treatment, after an average follow-up of 27 months, 96% of patients showed significant improvement in clinical symptoms. MMPRT biomechanically disrupt normal mechanics and lead to joint overload and development of SIFK; arthroscopic meniscal repair restores joint loading and improves patient prognosis (85).

#### 3.2.2 Core decompresion (CD)

CD reduces the intraosseous pressure in the necrotic area through drilling, also improves the blood circulation in the necrotic area, facilitates the nutrient supply to the bone and promotes the physiological healing of the bone. Duany NG et al. (86), in order to investigate the efficacy of joint-preserving surgery in the treatment of SIFK. They performed CD in 7 patients and achieved successful clinical outcomes in 5 patients. This procedure can be supplemented with bone grafting to prevent possible bone collapse due to core decompression. Deie M's team (87) applied CD with artificial bone implantation to 12 patients with SIFK, and the postoperative follow-up results were encouraging.

#### 3.2.3 Biomaterial implant technology (BIT)

BIT can rebuild mechanically durable articular bone plates and articular cartilage. Bone grafting and osteochondral transplantation are commonly used in the treatment of SIFK. Bone grafting provides a certain load bearing capacity for the weight-bearing area. Isolated bone grafting has been reported to have better long-term clinical outcomes than microfracture in the treatment of SIF in weight-bearing areas (88). However, bone grafting is more suitable for younger patients and those with smaller areas of necrosis. For patients with larger lesions, osteochondral transplantation is more advantageous. Tirico LEP et al. (89) followed up 7 SIFK patients with large lesions of medial femoral condyle treated by osteochondral allograft transplantation for at least 4 years. Results in good efficacy, durability and satisfaction in Koshino classification 2 and 3 SIFK patients who failed conservative treatment. Therefore, osteochondral transplantation in the treatment of subchondral bone collapse has shown excellent clinical efficacy both before and after collapse.

#### 3.2.4 Osteotomy

High Tibial Osteotomy (HTO) achieves direct reduction of medial compartment pressure through precise lower limb alignment correction. This biomechanical mechanism has been validated by three-dimensional finite element analysis, demonstrating a 40%–60% reduction in medial tibial plateau stress postoperatively. The stress-shielding effect at the osteotomy site can be further optimized using novel elastic plates (90). In a study by Takeuchi R (91) involving 30 SIFK patients subjected to HTO, full weight-bearing was achieved within 2 weeks postoperatively, with significant improvement in Knee Society Score (KSS). Biological studies (92) revealed that patients with SIFK exhibited markedly decreased concentrations of chemoattractants following HTO. This finding aligns with the “mechanical-metabolic” comorbidity theory of SIFK, providing novel insights into HTO's systemic regulatory effects beyond mere mechanical correction. Finite element modeling indicates that while proximal fibular osteotomy (PFO) reduces medial compartment stress by 30%–50% through disruption of fibular support to the lateral tibia, it concurrently increases lateral stress by 15%–20%, potentially accelerating lateral meniscal degeneration (93). This contrasts with HTO's “uniform decompression” effect, suggesting PFO is more suitable for mild-to-moderate varus (varus angle <10°) with intact lateral cartilage. Compared to HTO, current evidence on PFO for SIFK remains limited. HTO has emerged as the mainstream SIFK treatment due to its precise mechanical correction and anti-inflammatory effects, though optimization of fixation materials and complication management is warranted. PFO, as a simplified alternative, shows clinical potential but requires SIFK-specific studies to validate long-term safety. Future research should focus on the “mechanical-biological crosstalk” mechanism, leveraging smart implants and personalized surgical planning to achieve precision and minimally invasive joint preservation.

#### 3.2.5 Subchondroplasty (SCP)

SCP is a minimally invasive technique involving the injection of flowable synthetic materials into subchondral bone defects to enhance structural integrity, induce osteogenesis of healthy cancellous bone, and provide mechanical support to articular surfaces (94). Initially developed for osteoarthritic bone disorders, a retrospective study by Fitzpatrick et al. involving 11 patients with distal femoral subchondral defects demonstrated SCP's efficacy in pain relief, functional improvement, complication mitigation, and prevention of articular collapse (95). The core advantage of SCP lies in its dual “mechanical support–biological regeneration” mechanism: (1) Calcium phosphate cement with high compressive strength immediately stabilizes subchondral microfractures, halting collapse progression; (2) Its controlled degradation properties concurrently guide autologous bone ingrowth, avoiding stress shielding effects associated with metal implants. However, challenges persist in SCP application for SIFK). Intraoperative cement leakage rates may reach 8.7%, particularly with inadequate navigation precision, potentially triggering inflammatory synovitis. More critically, mismatched degradation kinetics and osteogenesis rates may create a mechanical vulnerability window (6–12 months postoperatively), elevating secondary collapse risks (96). As SCP remains understudied for SIFK, multicenter RCTs focusing on long-term biomechanical outcomes and skeletal structural evolution are warranted. Current patient selection criteria should be stringent, limited to: a. Early-stage (Stage I/II) SIFK; b. Non-collapsed lesions (<2 mm depth); c. Normal bone metabolism markers.

#### 3.2.6 Knee arthroplasty

**Figure 6:**
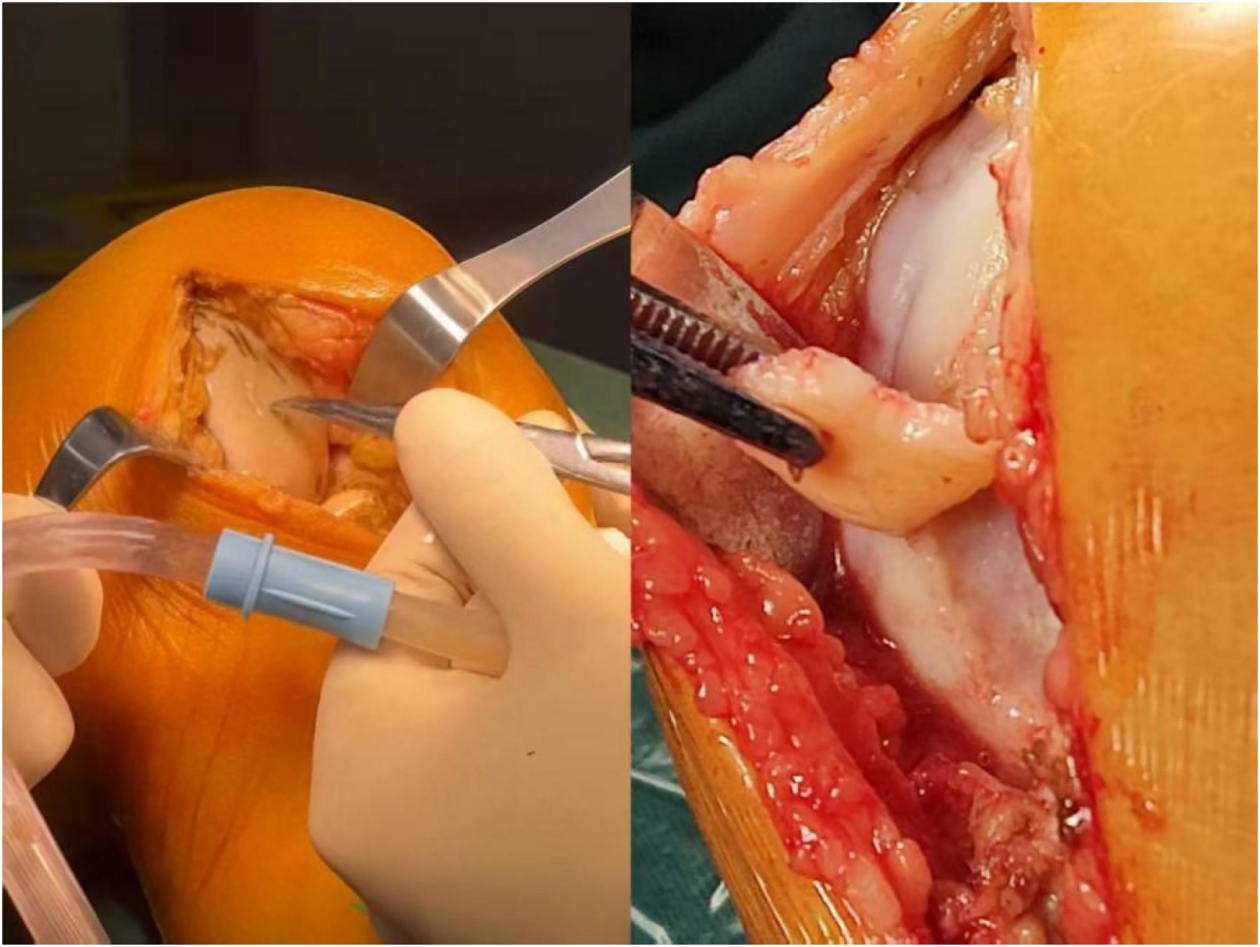
Intraoperative photograph: subchondral bone collapse of the medial femoral condyle.

The study by Pareek et al. (97) demonstrated rapid progression of SIFK, with a high surgical conversion rate. The 3-year surgical conversion rate reached 34%, of which joint arthroplasty accounted for 30%. When SIFK progresses to end-stage (Stage III/IV), characterized by persistent severe pain, functional impairment, and radiographic evidence of subchondral plate fracture, articular surface collapse (>2 mm), or progressive worsening of collapse, joint arthroplasty (UKA or TKA) becomes the necessary treatment option after failure of comprehensive non-surgical management (Figure 6). Pan et al. (98) proposed that patients with the following conditions would have poor prognosis with conservative treatment and should be considered for early joint arthroplasty: a. Necrotic area >5 cm²; b. Necrotic area exceeding 40% of the condyle; c. Relative extrusion percentage of medial meniscus ≥33% (with or without concomitant medial meniscus injury/subchondral bone marrow edema); d. Necrotic depth >20 mm on MRI (measured as the anteroposterior diameter of the necrotic area in sagittal view); e. Varus malalignment >6° in lower limb mechanical axis. Additionally, Lotke et al. (46) found that among patients with lesions involving 32% of the medial femoral condyle, only 6 out of 23 knees required surgical intervention, whereas over 50% of patients with medial femoral condylar necrosis involving more than half of the condyle ultimately required prosthetic joint replacement.

Saccone L et al. (99) conducted a systematic review of UKA for SIFK, incorporating 19 studies with 717 cases. The results reported that UKA exhibited acceptable prosthesis survival rates and revision rates during both short- and long-term follow-up, along with favorable clinical outcomes. Furthermore, Flury's team compared the efficacy of UKA vs. TKA in treating unilateral end-stage SONK. Their findings indicated superior postoperative recovery with UKA, whereas TKA demonstrated lower rates of prosthesis loosening and revision (100). National registry data revealed that the revision rate for UKA was threefold higher than that for TKA (101). Given that SIFK lesion size and surrounding bone quality may influence prosthesis fixation outcomes, the favorable results of UKA are contingent upon strict indication criteria and patient selection. UKA is only recommended for cases where the lesion is confined to a single compartment with intact ligaments and adequate bone quality. Conversely, TKA should be performed for Koshino stage IV patients presenting with concomitant patellofemoral osteoarthritis, valgus deformity, or bicompartmental lesion involvement that compromises unicompartmental prosthesis stability. Therefore, the selection between UKA and TKA requires comprehensive evaluation of lesion characteristics, overall joint condition, and individual patient factors. UKA represents an excellent option when strict indications are met, offering superior functional outcomes and faster recovery. Conversely, TKA remains indispensable for addressing complex lesions, multicompartmental involvement, or when long-term stability is prioritized. Given the current lack of definitive evidence regarding the impact of lesion characteristics (including depth, width, and extent) on the choice between UKA and TKA, future multicenter, large-sample RCTs are warranted. Such studies would optimize surgical decision-making and facilitate more precise, individualized treatment for patients.

## 4 Conclusion

SIFK, a disease with incompletely elucidated pathophysiology yet rapid progression, has long posed dual clinical challenges of delayed diagnosis and overtreatment. Through a systematic review of existing studies, this article clarifies that the core pathogenic mechanisms of SIFK have evolved from the early singular “osteonecrosis hypothesis” to a multidimensional model encompassing “abnormal bone metabolism–vascular impairment–meniscal biomechanical imbalance–iatrogenic/systemic factors”. In the therapeutic domain, SIFK has established a “staged strategy based on lesion size and disease progression”. Despite significant advancements in diagnosis and treatment, three critical issues remain unresolved: (1). Insufficient causal validation of pathophysiology—current studies predominantly rely on radiological correlation analyses, lacking direct pathological evidence; (2). Standardization gaps in treatment plans—such as the absence of an individualized dosing model for bisphosphonates and the undefined optimal therapeutic targets for PRP; (3). Scarce long-term prognostic data—with limited evidence on 10-year survival rates of joint-preserving surgeries and prosthetic longevity. Future research should focus on a closed-loop exploration of “mechanism–efficacy–prognosis”.
